# Telemedicine of patients with cystic fibrosis during the COVID-19 pandemic

**DOI:** 10.1590/1984-0462/2022/40/2021118IN

**Published:** 2022-05-06

**Authors:** Rafaella Lima Ferreira Costa, Rebeca Ferreira Costa, Christine Pereira Gonçalves, Renata Wrobel Folescu Cohen, Nelbe Nesi Santana

**Affiliations:** a Instituto Nacional de Saúde da Mulher, da Criança e do Adolescente Fernandes Figueira, Rio de Janeiro, RJ, Brazil.

**Keywords:** Cystic fibrosis, Telemedicine, COVID-19, Fibrose cística, Telessaúde, COVID-19

## Abstract

**Objective:**

To describe then experience of implementing routine teleconsultations in respiratory physiotherapy at a reference center for Cystic Fibrosis (CF) in Rio de Janeiro / Brazil, during the COVID-19 pandemic.

**Methods:**

Cross-sectional, descriptive, study with children and adolescents with CF. The sample was divided between participants and those who did not participate in the teleconsultations. The teleconsultations were multidisciplinary and carried out by videoconference or telephone, depending on the patient’s availability. The sequence of care provided by the team was organized together with the professionals, so that everyone could carry out individual and sequential teleconsultations. Physiotherapy appointments were divided into two segments: teleconsultation and telemonitoring. Demographic and clinical data were collected.

**Results:**

Among the 184 patients assisted in the center, 153 (83.2%) participated in the teleservices and, of these, 33 (21.6%) required telemonitoring; 31 (16.8%) patients did not participate in the teleconsultations for not answering the calls. There was no statistical difference between the group that participated or not in teleservices, nor among those who participated in teleconsultations and telemonitoring. The mean age of the studied population was 7.0±0.4 years. Regarding the CFTR gene mutation, 64.7% had at least one F508del allele and 30.9% of the sample had no pathogens in the sputum test.

**Conclusions:**

Most participants with CF participated in teleconsultations, highlighting the importance of remote assistance activities during the COVID-19 pandemic period. This strategy was considered as positive, and it may become permanent in the care of patients with CF.

## RESUMO

**Objetivo:**

Descrever a experiência de implantação da rotina de teleconsultas na fisioterapia respiratória de um centro de referência para fibrose cística (FC) no Rio de Janeiro, Brasil, durante a pandemia da COVID-19.

**Métodos:**

Estudo transversal descritivo com crianças e adolescentes com FC. A amostra foi dividida entre os participantes e aqueles que não participaram das teleconsultas. As teleconsultas foram multidisciplinares e realizadas por videoconferência ou telefone, conforme a disponibilidade do paciente. A sequência de atendimentos da equipe foi organizada em conjunto com os profissionais, de maneira que todos pudessem realizar as teleconsultas individuais e sequenciais. Os atendimentos de fisioterapia foram divididos em dois seguimentos: teleconsulta e telemonitoramento. Coletaram-se, dos prontuários, dados demográficos e clínicos.

**Resultados:**

Dos 184 indivíduos atendidos, 153 (83,2%) participaram dos teleatendimentos e, deles, 33 (21,6%) necessitaram de telemonitoramento; 31 (16,8%) não participaram das teleconsultas por não atenderem aos chamados. Não houve diferença estatística entre o grupo que participou dos teleatendimentos e aqueles que não participaram, nem entre os que participaram das teleconsultas e os que participaram do telemonitoramento. A média de idade da população estudada foi de 7,0±0,4 anos. Em relação à mutação do gene CFTR, 64,7% apresentavam pelo menos um alelo F508del e 30,9% da amostra não apresentou patógenos no exame de escarro.

**Conclusões:**

A maioria dos indivíduos com FC participou das teleconsultas, o que evidencia a importância das atividades de assistência remota no período da pandemia por COVID-19. Considerou-se que essa estratégia é positiva e pode tornar-se permanente no cuidado a pacientes com FC.

## INTRODUCTION

Cystic fibrosis (CF) is a serious, multisystemic, chronic, and progressive genetic disease, whose greatest involvement is observed in the respiratory system, where the tracheobronchial secretion is thicker and difficult to be remove. Retention of secretions in the respiratory system causes inflammation and accumulation of pathogens, favoring recurrent infections and worsening lung function.^1^ Given the complexity and the multisystemic component of the disease, the treatment needs to include numerous aspects, with an extensive daily routine consisting of oral and inhaled medications, specific diet, and physical therapy sessions.^2^ Respiratory physical therapy plays an important role in the routine of individuals with CF, and its daily practice reduces the progression of worsening pulmonary function and the number of days in the hospital, improving expectancy and quality of life of these patients.^3^

With the onset of the COVID-19 pandemic in Brazil, consultations and elective exams performed by patients with chronic disease were interrupted or postponed due to the virus’s dissemination power and the hitherto unknown impact on the chronically ill population, especially with lung manifestations. With the imposition of quarantine by the authorities, remote health care activities (telemedicine) were regulated by various bodies and class councils. The Federal Council of Physical Therapy and Occupational Therapy (Resolution No. 516, of March 20, 2020) established the possibility of planning physical therapeutic practices through non-face-to-face instruments, reducing possible negative repercussions of the suspension for a prolonged period of regular care provided.

One of the essential functions of a referral center is to provide adequate assistance to the population of patients with the disease in question. The Fernandes Figueira National Institute for Women, Children and Adolescent Health (*Instituto Nacional de Saúde da Mulher, da Criança e do Adolescente Fernandes Figueira* – IFF), in view of the quarantine, reinforced the need to transform face-to-face monitoring into remote monitoring, establishing a routine of multiprofessional telemedicine consultations. Among the active categories, the physical therapy clinic has also established a routine of telephone calls for this group of patients. The objectives of this study were to describe the experience of implementing routine telemedicine in respiratory physical therapy at a reference center in Rio de Janeiro, Brazil, as well as to characterize the population of CF patients who participated in these teleconsultations during the COVID-19 pandemic.

## METHOD

A cross-sectional, descriptive, and analytical study was carried out at IFF, considered a pediatric reference center for the treatment of CF in the state of Rio de Janeiro, Brazil. All children and adolescents aged between zero and 18 years with a CF diagnosis confirmed by the sweat test and/or by the presence of two mutations in the CFTR gene were included in the study, according to the consensus of the Cystic Fibrosis Foundation,^4^ followed up at the institute between March and December 2020. There were no exclusion criteria for the study.

Variables related to demographic (gender and age) and clinical (forced expiratory volume in one second — FEV_1_, bacterial colonization, and type of CFTR gene mutation) characteristics were obtained from the medical records. The sample was divided between individuals who participated in telemedicine and those who were not assisted by it. Those who were not assisted by telemedicine due to virtual absenteeism were followed up in person. Age was categorized, according to the World Health Organization, in children and adolescents.^5^ Regarding the type of CFTR gene mutation, participants were classified as homozygous and heterozygous for the F508del mutation and heterozygous for the other mutations. Bacterial colonization was segregated according to the referral center’s categorization as negative, colonized by Staphylococcus aureus (SA), non-mucoid Pseudomonas aeruginosa (NMPA), mucoid Pseudomonas aeruginosa (MPA), methicillin-resistant Staphylococcus aureus (MRSA) and Complex Burkholderia cepacia (BCC). FEV_1_ was analyzed according to the availability of the exam in the medical records of the sample participants. Spirometry results obtained between September 2019 and March 2020 were accepted.

Telemedicine was available every three months to all patients followed up at the referral center, but some caregivers preferred the in-person outpatient modality, not participating in the teleassistance. The teleconsultations were multidisciplinary, with the various specialties covering the treatment of CF, and carried out by videoconference on Skype^®^ or by telephone, depending on the patients’ availability. Among the specialties participating in the call center were pulmonology, physical therapy, nutrology, nutrition service, nursing, and social service. The appointment was made by the pulmonology service of the reference center and passed on to the other members of the multidisciplinary team. In addition, the sequence of care provided by the team was organized together with the professionals, so that everyone could carry out individual and sequential teleconsultations with the patients.

Physical therapy telemedicine was divided into two segments, as specified by the Ministry of Health: teleconsultation and telemonitoring. It is understood that teleconsultation is the conduction of consultation by a healthcare professional at a distance, with interaction between healthcare professional and patient; and that telemonitoring is the remote monitoring of health and/or disease parameters of patients using communication and information technologies, including the collection of clinical data from the patient, its transmission, processing, and management by a health professional, through an electronic system.^6^

Soon, following the multiprofessional schedule, the physical therapist performed the teleconsultation and, if necessary, started the telemonitoring. Among the reasons for carrying out telemonitoring were the patients’ or caregivers’ difficulty in assimilating the content offered in the teleconsultation and clinical worsening in the days preceding the virtual service. In addition to the scheduled teleconsultation, the demand for the patient or caregiver was instituted, when the latter called the professional and requested an extra teleconsultation if deemed necessary.

Among the topics covered in the physical therapy teleconsultation were treatment adherence, the proper use of prescribed drugs, the possibilities of performing techniques, and the use of physical therapy equipment for each patient individually. Thus, the possibility of open dialogue between the physical therapist and the family was offered, addressing important issues of the daily treatment routine, with the possibility of adapting it during the COVID-19 pandemic.

The data obtained were tabulated using the Microsoft Excel^®^ software and transferred to the Statistical Package for Social Sciences (SPSS) 25.0^®^ software for statistical analysis. The Kolmogorov-Smirnov test was performed to check the normality of the data, the Student’s *t*-test for independent samples to compare the means of continuous variables in the groups that participated or not in teleconsultation or telemonitoring, and the chi-square test for comparison proportions of categorical variables between these groups. The project was submitted and approved by the Ethics Committee for Research with Human Beings of IFF/Fiocruz by Certificate of Presentation for Ethical Assessment (*Certificado de Apresentação para Apreciação Ética* – CAAE) No. 34089320.9.0000.5269 and Opinion No. 4.209.436.

## RESULTS

The sample consisted of 184 individuals, 137 children and 47 adolescents. The mean age of the population studied was 7.0±0.4 years. Among the participants, 47.3% were male. Regarding the CFTR gene mutation, 64.7% had at least one F508del allele. When analyzing sputum colonization, 30.9% of the sample did not present pathogens in the bronchial tree. Among the population studied, 67 children did not present recent spirometry in their medical records due to their age group.

Of the 184 individuals assisted at the reference center, 153 (83.2%) participated in the telemedicine service. Regarding the studied variables, only the distribution of bacterial colonization in sputum showed a statistically significant difference between the groups of participants and non-participants in the teleconsultations (p<0.05). Regarding the value of FEV_1_, although the mean observed among the participants who did not receive telemedicine services was higher, there was no statistically significant difference when compared to those who were assisted by such services (Table 1).

**Table 1.** Description of demographic and clinical variables of patients with cystic fibrosis in relation to telemedicine care.

Teleconsultation				
	Total (n=184)	Yes (n=153)	No (n=310)	p-value
Male	87.0 (47.3)	71 (46.4)	16 (51.6)	0.700
Age (years)	7.2±5.3	7.0±0.5	8.0±1.0	0.330
Children	137.0 (74.4)	115.0 (75.1)	22.0 (70.9)	0.340
Adolescents	47.0 (25.5)	38.0 (24.8)	9.0 (29.0)	
% FEV_1_	78.5±20.5	77.6±21.1	83.3±17.3	0.270
Mutations				
F508del/F508del	39.0 (29.4)	37.0 (31.1)	2.0 (14.3)	0.390
F508del/other	47.0 (35.3)	42.0 (35.3)	5.0 (35.7)	
Other mutations	47.0 (35.3)	40.0 (33.6)	7.0 (50.0)	
Colonization				
Negative	69.0 (37.7)	47.0 (30.9)	22.0 (71.0)	<0.001*
SA	33.0 (18.0)	31.0 (20.4)	2.0 (6.5)	
NMPA	19.0 (10.4)	19.0 (12.5)	0	
MPA	23.0 (12.6)	18.0 (11.8)	5.0 (16.0)	
MRSA	17.0 (9.3)	15.0 (9.9)	2.0 (6.4)	
BCC	22.0 (12.0)	22.0 (14.5)	0

Variables expressed as n (%) or as mean±standard deviation. SA: Staphylococcus aureus; NMPA: non-mucoid Pseudomonas aeruginosa; MPA: mucoid Pseudomonas aeruginosa; MRSA: methicillin-resistant Staphylococcus aureus. BCC: Burkholderia cepacia complex. FEV_1_: forced expiratory volume in one second. *p<0.05.

Of the 153 individuals who were assisted by teleconsultation, 33 (21.6%) needed telemonitoring to better monitor their clinical conditions due to the worsening of the disease. There was no difference in clinical characteristics between those who participated by teleconsultation and those who required telemonitoring (p>0.05) (Table 1). 

## DISCUSSION

The COVID-19 pandemic stimulated the urgent structuring of a distance service. However, telemedicine can also be established as a permanent care strategy at the referral center and not only during the pandemic period, especially in situations of low adherence and clinical worsening without the need for hospitalization, as telemedicine incorporates a wide range of activities that go beyond patient care, also encompassing actions to promote health and education, among others.^7^

Of the patients seen at the referral center, 83.2% participated in teleconsultations, which shows us good acceptance and adherence to telehealth. This result is corroborated by Gur et al., who describe the use of teleconsultation as feasible and acceptable. In addition, the patients were willing to carry out the calls in their own environment, where they felt safe and comfortable. The authors also report that online “chat” improves communication with the team.^8^

The use of telemedicine in the COVID-19 pandemic showed positive acceptance by patients, increasing the accessibility of care. However, some challenges were detected regarding the acquisition of pulmonary function tests, cultures, laboratories, and imaging data.9 In addition, access to appropriate technologies is required for successful virtual care, and some patients may not have access to high-speed internet connectivity or internet-enabled devices, which restricts their participation in teleconsultations.^10^

Specialized referral centers with multidisciplinary care are associated with improved life expectancy in CF patients.^11^ In our study, they had a mean FEV_1_ of 78.4±20.5, and 64.7% of these children and adolescents presented at least one F508del allele. The mean value of FEV_1_ found categorically reflects mild obstructive disorder^12^, so that good clinical conditions can be inferred in the studied sample. Regarding pathogens colonized in sputum, although the predominance in both groups is of negative patients, there was a statistically significant difference in the distribution of bacterial colonization, perhaps due to the difference in sample size between the group that participated in telemedicine and those that did not. 

Although care at the referral center is very important, access to such appointments can be difficult for families who do not reside in large centers. Barriers to care that are related to distance can be partially reduced with the use of technologies.^13^ Teleconsultation and telemonitoring in CF have been described in the literature as a possibility of access to health care, regardless of the patients’ location. Furthermore, they can minimize the risk of cross-infection, as they reduce the frequency of hospital visits. It can be added that telemedicine can reduce the social impact, as parents are less absent from work and children from school. Some studies, even before the COVID-19 pandemic, had already evaluated the telephone service for the population of CF patients, as well as its assessment instruments. The authors, then, considered teleconsultation to be an effective alternative for accessing health information, in addition to face-to-face care.^14,15^

Among the facilitating points for telemedicine, the possibility for the patient to attend the multidisciplinary consultation at the reference center without moving from their own home was evidenced, which is especially important in pandemic times. In addition, the dynamics of the multidisciplinary team, referring patients from one professional to the other, optimized and reduced the waiting time between appointments. As barriers, some aspects of telemedicine made this type of care difficult, such as the need for technology, whether through devices or internet networks. Also, as an obstacle, we can mention the inability to perform exams exclusively in person, such as spirometry, radiography, and computed tomography.

Perkins et al. studied telemedicine patterns, attitudes, and preferences among physicians at seven CF referral centers. The authors noted that clinicians had concerns about components missing from routine assessment. However, although most professionals had no previous experience with telemedicine, it proved to be highly satisfactory and efficient and improved the doctor-patient relationship.^16^

Jaclyn et al. evaluated the understanding of people with CF and their families of the transition experience between face-to-face care and that provided by telemedicine in 11 reference centers. The authors realized that the second is a powerful tool that allowed continuous access to the care of patients with chronic diseases through the health system. Therefore, it will continue to be an important aspect of care for CF patients, who already had recommendations for social distancing. As limitations, the study presented the usual personal assessments, the lack of sputum culture and pulmonary function tests, which worried the participants.^17^

In the present study, it was observed that most children and adolescents with CF participated in teleconsultations and adhered to them, which demonstrates the importance of remote care activities during the period of the COVID-19 pandemic. This care strategy was considered positive by the team and can become permanent in CF care at the reference center in question, but further studies are needed to verify patient satisfaction, efficiency, and accessibility to this care option.
